# Dietary Diversity and Nutrient Intake of Han and Dongxiang Smallholder Farmers in Poverty Areas of Northwest China

**DOI:** 10.3390/nu13113908

**Published:** 2021-10-30

**Authors:** Zhuo Wang, Youhai Chen, Shihua Tang, Siqi Chen, Shaoqing Gong, Xinying Jiang, Liang Wang, Ying Zhang

**Affiliations:** 1 School of Public Health, Lanzhou University, Lanzhou 730000, China; zhwang2018@lzu.edu.cn (Z.W.); chenyh629@163.com (Y.C.); tangshh19@lzu.edu.cn (S.T.); chensq18@lzu.edu.cn (S.C.); 2 School of Public Policy and Administration, Xi’an Jiaotong University, Xi’an 710049, China; gongshaoqing@mail.xjtu.edu.cn; 3 Department of Health and Nutrition Sciences, Brooklyn College of the City University of New York, Brooklyn, NY 11210, USA; xinyinjiang@brooklyn.cuny.edu; 4 Department of Public Health, Robbins College of Health and Human Sciences, Baylor University, Waco, TX 76798, USA

**Keywords:** smallholder farmers, dietary diversity, nutrient adequacy ratio, ethnic minority, China

## Abstract

This study aimed to evaluate the status of dietary diversity and nutrient intake among Han and Dongxiang smallholder farmers in poor rural areas of northwest China. In this cross-sectional study, dietary intake was assessed in 499 smallholder farmers aged 18–75 years from two nationally designated impoverished counties in Gansu Province, China, using three consecutive 24 h dietary recalls. The dietary diversity score (DDS) and nutrient adequacy ratio (NAR) were adopted to assess dietary diversity and micronutrient adequacy, respectively. The mean DDS (range from 1 to 9) in participants was relatively low (3.81 ± 1.01). Consumption of grains was excessive, while consumption of vegetables, fruits, meat, beans, eggs, fish, and dairy was inadequate. The NAR values were higher in Han Chinese, with the exceptions of vitamin C, potassium, pyridoxine, and selenium (*p* < 0.05). For each nutrient, the high DDS group had a higher mean NAR (*p* < 0.05), except for pyridoxine. High household monthly income, being Han Chinese, high DDS, and being aged over 45 years were positively associated with mean adequacy ratio (MAR) of 14 micronutrients evaluated. Lack of dietary diversity and insufficient intake of essential micronutrients are public health concerns in northwest China. Nutrition education and other proper methods to address these issues are needed.

## 1. Introduction

Micronutrient deficiency, known as “hidden hunger”, is one of the most critical nutritional problems affecting the health of children and adults currently, as well as being one of the most prevalent public health issues worldwide [1]. While the dietary requirement for micronutrients is quite small, deficiencies can have negative impacts on health that will ultimately result in death if untreated [2]. Micronutrient deficiencies can not only affect the immune system and function, but also hamper development and result in metabolic disorders [3]. Some research suggests that dietary diversity has a favorable impact on micronutrient adequacy, and is positively associated with health status across age groups [4,5,6]. Some studies have documented that a diverse diet is associated with micronutrient adequacy, and leads to positive health outcomes [7,8,9]. A diverse diet also decreases the risk of being stunted and underweight [10,11]. Lack of dietary diversity is a serious problem globally, and is most prevalent in the poorest households [12]. In summary, it is critical to assess micronutrient adequacy and diet diversity in vulnerable populations.

It is estimated that more than 2 billion individuals around the world are suffering from micronutrient deficiencies, particularly vitamin A, iodine, iron, and zinc [2]. Furthermore, there is a high prevalence of micronutrient deficiency in developing countries [6,13]. China is one of the most economically dynamic countries, with complex lifestyles and dietary habits [1], in which major changes in the country’s population and socioeconomy have resulted in significant challenges to nutrition and health [6]. Undernutrition and micronutrient inadequacy are still concerning problems in China, especially among rural residents living in poverty [5]. Health-related illnesses resulting from energy and nutrient deficiencies, such as low body weight and anemia, are adverse consequences of the insufficient food supply and unbalanced dietary habits in low-income areas [14,15].

China is one of the largest developing countries that is experiencing significant transitions in multiple areas of society, including poverty levels and dietary habits [16]. From 2012 to 2019, the rural poor population in China declined from 98.99 million to 5.51 million [17]. Gansu Province lies in northwest China. It is predominantly an agricultural region, and had 31 impoverished counties until 2019 [18]. Smallholder farmers live in impoverished counties with insufficient infrastructure, development, medical and health services, and fresh fruits and vegetables. When food is available, many low-income households consume strictly cereal-based diets lacking in vegetables, fruits, and dairy, thus increasing the risk of micronutrient deficiency [19,20]. Both Dongxiang and Han Chinese are the dominant ethnic groups in the two regions studied. The Dongxiang Chinese, a distinctive minority group living in Gansu Province of China, are unique in their living environment, lifestyles, and dietary behaviors, compared to their extensive Han counterparts. The limited available evidence suggested that Han rural students grow faster in chest circumference, height, weight, and body mass index (BMI) than their Dongxiang counterparts [21], and blood lipids and BMI were significantly higher among the Dongxiang than Han Chinese [22]. This suggested that nutritional imbalance may be especially common in this group.

In addition to the adequacy of each nutrient, it is essential to assess the overall healthiness and diversity of the diet, since the diet contains a wide range of foods that provide a comprehensive profile of nutrients instead of individual nutrients or foods [11]. It has been reported that the dietary diversity score (DDS) among adults in southwest China was 5.2 (on a scale of 9) [23]. Some studies have explored the dietary diversity status of Chinese children and adults, the factors that influence dietary diversity, and the connection between micronutrient adequacy and dietary diversity [5,6,24]. Few studies, however, have investigated the association of DDS with micronutrient adequacy in Han and Dongxiang smallholder farmers, especially in low-income rural areas of northwest China. This study aimed to evaluate the dietary diversity and nutrient intake among Han and Dongxiang smallholder farmers, as well as to investigate the association between dietary diversity and nutrient intake. The study also explored associated factors with mean adequacy ratio (MAR) among farmers in low-income rural areas of Gansu Province in China.

## 2. Materials and Methods

### 2.1. Study Participants’ Nutrient Adequacy Ratio (NAR)

We collected the information from June to July 2020 in two counties in Dingxi city and Dongxiang Autonomous County, Gansu Province in northwest China. Both are poverty-stricken counties. Dongxiang Autonomous County, situated in Linxia Autonomous Prefecture, is the only autonomous county with Dongxiang Chinese as the main ethnic group in China. Dongxiang Chinese have high levels of illiteracy, and speak the distinctive Dongxiang language [22]. They commonly follow Islam. The economic development is lagging in the regions, which may lead to a lower supply of fresh foods and an unchanged food structure. Participants were recruited from the two counties using a stratified random sampling method. Two townships containing 7–8 villages were randomly selected from each county. In each village, 25–30 smallholder farmers were invited to participate in the study and sign the informed consent. Participants must have lived in the area for at least 6 months, be 18 years of age or older, and be able to give answers to relevant questions to be eligible. Altogether, we surveyed 512 participants. After excluding 13 participants with extreme energy intakes (above 4000 kcal/day or below 800 kcal/day) [25], serious illness, or missing important information, the final analysis was conducted on 499 participants with a valid questionnaire rate of 97.46%.

### 2.2. Dietary Data

Interviewers trained from Lanzhou University made visits to selected households to collect food consumption information through three consecutive 24 h dietary recalls. Briefly, the Retrospective Dietary Survey Ancillary Reference Food Atlas [26], with a photographic album of different food items, was used to determine portion size reported by the respondents, and then the average daily intakes of various food items, energy, and nutrients in diet were calculated using the software Nutrition (V. 2.7.7.6) based on the Chinese Food Composition Table. In addition, the socio-demographic characteristics of participants were recorded by questionnaires. The Cronbach test and the Kaiser–Meyer–Olkin (KMO) test were used for reliability and validity (Cronbach’s α = 0.744 and KMO = 0.845), respectively. Generally, the values of α and KMO more than 0.7 indicate good reliability and validity.

DDS was determined by accounting for the number of food groups consumed within three days, according to the dietary survey. In accordance with the latest Chinese Dietary Guidelines issued in 2016, all foods groups were classified into nine different categories: grains (comprising cereals, tubers and roots), fruits, vegetables, meat (comprising poultry, beef, pork, and organs), legumes (comprising nuts, beans, and seeds), fish (comprising freshwater fish, aquatic products, seafood), eggs, dairy products (comprising milk and milk products), and oil (comprising vegetable and animal oil) [5,23]. DDS was computed by adding together the quantities of different food groups consumed. For a food group, the all-inclusive DDS calculation approach was adopted with no minimum intake. A minimum of 0 and a maximum of nine points could be scored. A higher DDS means more diversity and a wider variety of food groups consumed [23].

### 2.3. Anthropometric Measures

Anthropometric measurements were collected by trained interviewers in accordance with the proposed protocol of the World Health Organization (WHO). While not wearing thick clothing, weight was weighed with a platform scale to an accuracy of 0.1 kg. While not wearing shoes or a hat, height was obtained to the nearest 0.1 cm with a portable stadiometer. Body mass index (BMI) was calculated as weight (kg) divided by the square of the height (m^2^), and was classified into four groups, according to Chinese criteria: obese (BMI ≥ 28), overweight (24 ≤ BMI < 27.9), normal weight (18.5 ≤ BMI < 23.9), and underweight (BMI < 18.5) [27].

### 2.4. Definitions of Study Variables

Age was divided into three groups, according to the WHO standards: young-aged 18–44, middle-aged 45–59, and senior-aged 60 and older [28]. Ethnic groups were categorized as Han and Dongxiang. Marital status included married and unmarried. Education level was divided into four groups: primary and less than primary, middle school, high school or secondary school, and college/technical college or above. Income was measured based on the last month’s household income. To determine the nutritional knowledge of participants, the survey included 15 multiple-choice questions (macronutrients: four, micronutrients: five, sources: four, functions: three) concerning macronutrients and micronutrients, as well as sources and functions of nutrients. Correct answers were given one point.

### 2.5. Assessment of Nutrient Adequacy

To estimate the nutritional adequacy of diet, NAR was calculated for 14 micronutrients: vitamin A, vitamin E, thiamine, riboflavin, pyridoxine, vitamin C, niacin, calcium, potassium, magnesium, iron, zinc, phosphorus, and selenium. The NAR value for a given nutrient is the ratio of the current intake of the nutrient of the respondent to the Estimated Average Requirement (EAR) at the individual level for the corresponding age group [29]. EAR values were derived from the most recent Chinese Dietary Reference Intakes (DRIs) [30]. Given that some nutrients such as vitamin E and potassium did not have an EAR value, Adequate Intake (AI) was used as an alternative basis for calculating NAR. Theoretically, NAR values may be less than, equal to, or greater than one for energy and nutrients [10]. MAR reflects overall dietary adequacy, and is assessed by dividing the sum of all NARs by the quantities of nutrients evaluated (n = 14). For both NAR and MAR, a value of one is desirable, as it implies that the intake covers the requirements.

### 2.6. Statistics Analysis

To improve the accuracy of data, it was all double entered independently by two separate people, using EpiData 3.1 (CDC, Atlanta, GA, USA). Descriptive statistics were taken to count basic information about the interviewees. The Shapiro–Wilk test was conducted to verify the normality of distribution of data. On the one hand, for normally distributed data (DDS), it was presented as mean and standard deviation (SD). On the other hand, the data that did not follow the normal distribution (NAR, MAR, energy, nutrients, and food groups) were expressed as median (interquartile range, IQR). To examine the differences in DDS scores between basic characteristics, Student’s *t*-test and one-way ANOVA were used for two-group comparisons and multiple comparisons, respectively. Furthermore, the Kruskal–Wallis test was employed to test the differences in NAR, MAR, energy, nutrients, and food groups across the tertiles of DDS (low, medium, and high), and the Mann–Whitney *U* test was used to examine the difference in NAR, MAR, energy, nutrients, and food groups between Han and Dongxiang Chinese. Moreover, to explore associations between NAR and DDS, Spearman correlation analysis was adopted for all the survey data. Finally, multiple linear regression was employed to evaluate the predictors of MAR. All data analyses were conducted using the Statistics Package for Social Sciences (SPSS) software program (version 24, SPSS Inc., Chicago, IL, USA). Unless otherwise stated, all statistical analyses were performed at the statistical significance level of 0.05 (two-tailed tests).

### 2.7. Ethics Statement

This study was approved by the Medical Ethics Committee of School of Public Health in Lanzhou University (NO: IRB20050901). All smallholder farmers who participated in the study were required provide informed consent before conducting the survey.

## 3. Results

### 3.1. Dietary Diversity Score (DDS) Based on Basic Characteristics

Smallholder farmers aged over 60, who had a higher education level, had a higher household monthly income, had household size of 3~5, and were overweight had a significantly higher DDS (both *p* < 0.01). Participants who were Han Chinese and had nutritional knowledge above the mean had a significantly higher DDS than those who were Dongxiang Chinese and had nutritional knowledge below the mean, respectively (4.18 vs. 3.45, 4.39 vs. 4.26, both *p* < 0.001) (Table 1).

### 3.2. Energy, Nutrients, and Food Groups Intake of the Participants by Ethnicity Groups

Participants’ median dietary intakes of vitamin A, riboflavin, pyridoxine, vitamin C, and calcium were extremely low, not meeting one quarter or half of the Estimated Average Requirement (EAR). Compared with Dongxiang Chinese, Han participants had higher cholesterol, fat, protein, and energy intake (all *p* < 0.05). Moreover, micronutrients (vitamin A, vitamin E, thiamine, riboflavin, niacin, and zinc) intakes were higher in Han than in Dongxiang participants (all *p* < 0.05). However, Dongxiang smallholder farmers had higher pyridoxine, vitamin C, potassium, and selenium intakes. According to the Chinese Dietary Guidelines, grain intake was excessive, and intake of the other eight food items was inadequate. More specifically, the median dietary intake of the other seven food groups besides grains and oil were not adequate in the adults. Consumption of multiple food groups such as fruits, vegetables, meat, fish, eggs, beans, and oil in Han participants were higher than those in Dongxiang participants (all *p* < 0.05) (Table 2).

### 3.3. Consumption of Energy, Nutrients and Food Groups Based on the Dietary Diversity Score (DDS) Groups

In the present study, 499 respondents had a normal distribution of DDS ranging from two to eight, meaning that no participant consumed nine food groups, and no participant consumed less than two food groups. Respondents were categorized into three groups: low, medium, and high. The low DDS group involved those with a DDS range of 2–4, the medium DDS group involved those with a DDS equal to 5, and the high DDS group involved those with a DDS range of 6–8.

Of the four macronutrients assessed, significant differences existed between the groups. All macronutrients apart from carbohydrates increased as DDS increased. Of the fourteen micronutrients assessed, eight differed significantly between the DDS groups, and the intakes of vitamin A, vitamin E, riboflavin, niacin, calcium, zinc, and selenium increased with DDS. There were also significant differences in intakes between the DDS groups for all food groups. In multiple comparisons, all nutrients or food groups were significantly different, and had higher consumption levels for medium and high DDS group versus low DDS group, with the exceptions of carbohydrates, vitamin B_6_, vitamin C, and grains. Although consumption of grains exceeded recommended intake, people with high DDS group consumed little fish, dairy, vegetables, fruits, meats, and beans, not reaching the recommended intake (Table 3).

### 3.4. Nutrient Adequacy Ratio (NAR) of Specific Nutrients in Two Ethnicity Groups

The NAR values of all micronutrients, with the exceptions of vitamin C, selenium, potassium, and pyridoxine, were higher in the Han Chinese. The NAR values for calcium, pyridoxine, and vitamin A were the lowest, not reaching a quarter of their EAR in the participants. The overall mean NAR of vitamin A was 0.088 (0.028, 0.251) (ranging from 0.044 in Dongxiang to 0.154 in Han Chinese) and the overall mean NAR of calcium was 0.231 (0.176, 0.303) (ranging from 0.228 in Han to 0.236 in Dongxiang Chinese) (Table 4).

### 3.5. Nutrient Adequacy Ratio (NAR) of Specific Nutrients by Dietary Diversity Score (DDS) Groups

Of the 14 micronutrient NAR values assessed, all but those for thiamine, vitamin C, potassium, and pyridoxine increased with DDS. Moreover, MAR values were significantly higher in the high DDS group. Spearman correlation coefficients for each NAR of specific nutrients associated with DDS were determined for all participants. The NAR of micronutrients, except for thiamine, pyridoxine, vitamin C, iron, potassium, and magnesium, were significantly positively correlated with DDS (*r* = 0.170, *p* < 0.001) (Table 5).

### 3.6. Linear Regression Model of Predictors of Mean Adequacy Ratio (MAR)

In the multivariable linear regression model (Table 6), the participants who had a household monthly income ≥ 3500 (*β* = 0.116, 95%CI: (0.012, 0.219)), high DDS (*β* = 0.128, 95%CI: (0.019, 0.238)), age over 45 (*β* = 0.068, 95%CI: (0.006, 0.130)), and being Han Chinese (*β* = 0.084, 95%CI: (0.014, 0.155)) were positively associated with MAR, while being female (*β* = −0.140, 95%CI: (−0.196, −0.083)) was negatively associated with MAR.

## 4. Discussion

The present study assessed the dietary diversity and nutrient adequacy among smallholder farmers in Gansu Province. The DDS, based on nine food groups, was adopted to assess dietary diversity. NAR and MAR indicators were used to measure the probability of nutritional adequacy. Additionally, the present study demonstrated that dietary diversity was positively associated with most dietary micronutrient intake.

The mean DDS was 3.81 in this study, which was lower than the DDS reported in other studies (5.20 in Zhang et al., 4.46 in Yin et al. [23,31]). Variations in the results of DDS may arise from the different study samples and different measurements of DDS [32]. Yin et al. conducted their study among elderly people, partly from urban areas [31], where it is more convenient to access a variety of foods than in rural areas. In the present study, participants were from rural areas, where access to diverse foods was more costly than in urban areas [33]. Studies have also reported that DDS was directly related to income [34,35,36], supporting the present study’s findings that the smallholder farmers’ DDS differed based on different household monthly incomes. The majority of participants were smallholder farmers with very low incomes, and thus, most participants had a lower DDS in the present study, which is in line with the findings of previous studies [37,38,39]. Some studies have demonstrated that lower education levels predict lower DDS [23,40,41], and our study concluded that most of the population born in the 1950s–1970s in areas of poverty had limited opportunity to receive a high level of formal education.

We also found that Han Chinese appeared to have higher odds of DDS than Dongxiang Chinese, which is consistent with a previous study among adults in southwest China [23]. This suggests that Dongxiang smallholder farmers have a less diverse diet than their Han peers. No significant difference between DDS and sex was found in our study, which is inconsistent with a previous study [23,42], possibly since there were more males than females in the present study. In this study, we also found that DDS significantly differed by weight status. Previous studies have shown that obese individuals have higher DDS values than normal-weight individuals [34,42], and the present study reported a higher DDS among overweight participants than in those who were underweight and normal weight. A possible explanation is that individuals with higher DDS are likely to have unhealthy diets [33]. Smallholder farmers are less nutritionally literate, and some of them consume adequate types of food but also excessive amounts simultaneously, thus indicating that dietary interventions and nutrition education are necessary.

Imbalanced dietary practices are more common in underdeveloped areas of Gansu Province. A monotonous diet usually means unhealthy eating habits, and some studies have related the DDS to higher rates of obesity [23,43] and poor nutrient adequacy [44,45].

The adequacy rates for some micronutrients, such as calcium, vitamin A, vitamin C pyridoxine, and riboflavin, in smallholder farmers were low. The imbalance of micronutrients may have a large burden on smallholder farmers’ health, increasing the risk of chronic diseases. Among the nutrients assessed in this study, deficiency in vitamin A intake was the most common. Given that vitamin A has a major role in multiple aspects of health, such as immune function and eye and skin health, dietary interventions to address the insufficient intake of this nutrient should be implemented, such as increasing intake of organic and dark green vegetables [46]. Moreover, deficiency in riboflavin and pyridoxine results in homocysteine accumulation, which is related to cardiovascular disease, hypertension, and cognitive impairment [6]. Vitamin B complex intake deficiency may be related to low intake of whole grains, legumes, vegetables, fruits, and some animal foods [47]. Furthermore, dairy products are the most abundant origin of calcium, yet they are consumed at relatively low level, especially in rural areas of poverty in northwest China [48]. Vitamin C is a potent water-soluble antioxidant that not only protects immune cells in the innate system by fighting ROS and regenerating oxidized glutathione and vitamin E, but also regulates B and T-lymphocyte differentiation and proliferation through gene regulation in adaptive immunity [49]. Participants’ vitamin C intake, however, was inadequate in the present study. Therefore, it is necessary to increase vegetable and fruit intake. We also found that out of the 14 micronutrients assessed, ten were significantly different between the Han and Dongxiang Chinese, suggesting that dietary interventions for ethnic minorities should be enhanced in the future.

In addition, a positive correlation was also found between DDS and NAR in most micronutrients, which is in accordance with previous findings [5,23,50]. Zhang et al. estimated the dietary diversity and nutrient profile of adults in southwest China, and showed a positive correlation between NAR values of most nutrients and DDS. Meng et al. demonstrated a positive association between DDS and nutrient adequacy in children aged 3–17 years in China. Similarly, higher DDS was directly associated with adequate nutrient intake. Participants with high DDS in the present study had higher adequacy rates for intake of most micronutrients (vitamin A, vitamin E, riboflavin, niacin, calcium, zinc, phosphorus, and selenium), with Pearson correlation coefficients in the range of 0.15 to 0.71. Moreover, the DDS was associated with the MAR, showing a significant increase in the high DDS group, which is consistent with a previous study [29]. Despite this, the high DDS group did not perform well for vitamin A, calcium, and pyridoxine intake, which showed that mean NARs were below 50% of EAR. These findings emphasize the need for increasing food diversity to acquire adequate nutrients. Consuming a variety of food groups including fruits, vegetables, dairy, and other healthy food groups will ensure adequate intake of nutrients [45,51]. Given that essential nutrient content varies among foods, the dietary guidelines suggest that people should consume a variety of foods.

To the best of our knowledge, the present study is the first to assess the relation of dietary diversity status and nutrient adequacy in poor rural areas of northwest China. Most of the previous studies are primarily focused on the dietary diversity of children and urban areas, but not on individuals living in poor and remote rural areas, especially among Han and Dongxiang smallholder farmers. Our study fills the gap by providing a comprehensive evaluation of dietary diversity, nutrient adequacy, and their relation in remote rural areas in Gansu Province. An important strength of this study was the use of paired sampling in Han and Dongxiang Chinese, allowing us to compare the dietary diversity and nutrient intakes in these two ethnic groups.

This study also has some limitations. First, a cross-sectional study could not determine causality. Second, it was conducted only in two counties of one province, and the data were limited to rural populations, thus it does not represent the urban populations. Additionally, the sample size was small, so the findings may not be generalized to a large population. Third, the three-day dietary recall also had limitations in terms of assessing nutrients and due to the absence of biochemical tests, we could not offer more precise results on micronutrient levels. Fourth, the language barrier of the Dongxiang Chinese may have affected the accuracy of the information collected by the questionnaire.

## 5. Conclusions

This study indicated that the dietary diversity status and micronutrient intake of smallholder farmers in poor rural areas of northwest China were relatively low. In particular, Dongxiang Chinese were more vulnerable to micronutrient deficiency compared to Han Chinese. Additionally, dietary diversity was found to be positively associated with nutrient intake. Efforts need to be taken to maximize dietary diversity, and increased consumption of fruits, vegetable, and dairy is encouraged. Furthermore, low income and limited nutritional knowledge level were associated with low adequacy of micronutrient intake. Nutritional education and policies to promote dietary diversity and to increase micronutrient intakes are necessary.

## Figures and Tables

**Table 1:** Dietary diversity scores and basic characteristics of participants (*N* = 499).

Basic Characteristics	N	(%)	DDS (Mean ± SD)	*p*
Gender				
Male	330	(66.13)	3.82 ± 0.99	0.891
Female	169	(33.87)	3.80 ± 1.07	
Ethnicity				
Han	250	(50.10)	4.18 ± 1.10	<0.001 ***
Dongxiang	249	(49.90)	3.45 ± 0.76	
Age (years)				
18~	205	(41.08)	3.85 ± 1.05	0.002 **
45~	216	(43.29)	3.91 ± 1.02	
60~	78	(15.63)	3.45 ± 0.82	
Education level				
Primary or below	363	(72.75)	3.59 ± 0.92	<0.001 ***
Middle school	82	(16.43)	4.22 ± 0.96	
High school/secondary technical school	35	(7.01)	4.43 ± 1.04	
College/technical school	19	(3.81)	5.21 ± 0.86	
Household monthly income (yuan)				
<1500	324	(64.93)	3.60 ± 0.91	<0.001 ***
1500~	97	(19.44)	3.98 ± 1.01	
2500~	38	(7.62)	4.29 ± 1.11	
≤3500	40	(8.01)	4.68 ± 1.05	
Household size				
<3	36	(7.21)	3.39 ± 0.80	<0.001 ***
3~	259	(51.90)	4.04 ± 1.11	
6~	173	(34.67)	3.65 ± 0.85	
≥9	31	(6.22)	3.35 ± 0.71	
Marital status				
Married	452	(90.58)	3.82 ± 1.01	0.855
Unmarried	47	(9.42)	3.79 ± 1.04	
Nutritional knowledge				
Above the mean	211	(42.28)	4.39 ± 0.81	<0.001 ***
Below the mean	288	(57.72)	4.26 ± 1.10	
Weight status (determined by BMI)				
Underweight	38	(7.62)	3.58 ± 0.92	0.006 **
Normal weight	312	(62.53)	3.76 ± 1.02	
Overweight	117	(23.45)	4.09 ± 1.01	
Obesity	32	(6.40)	3.63 ± 0.91	

DDS: dietary diversity score. SD: standard deviation. BMI: body mass index. *p* value was calculated using Student’s *t*-test or one-way ANOVA. ** *p* < 0.01, *** *p* < 0.001.

**Table 2:** The daily consumption of energy, nutrients, and food groups among participants by ethnicity groups (*N* = 499).

Nutrients	Total (*N* = 499)	Han (*n* = 250)	Dongxiang (*n* = 249)	*p*
Energy	1784.00 (1401.33, 2194.00)	1893.83 (1477.42, 2256.25)	1696.33 (1352.50, 2152.17)	0.042 *
Protein	50.37 (39.73, 63.63)	51.42 (42.00, 64.97)	47.63 (37.00, 62.90)	0.044 *
Fat	31.73 (25.00, 42.13)	37.00 (29.23, 51.57)	28.47 (22.53, 33.13)	<0.001 ***
Carbohydrates	312.10 (241.23, 396.76)	315.13 (242.41, 389.60)	307.00 (240.77, 410.03)	0.826
Cholesterol	5.67 (0.00, 151.67)	65.33 (0.00, 257.33)	0.00 (0.00, 32.50)	<0.001 ***
Vitamin A	46.33 (15.00, 132.33)	82.67 (32.67, 185.50)	23.00 (9.17, 64.50)	<0.001 ***
Vitamin E	18.23 (14.72, 22.57)	20.27 (15.50, 25.64)	17.12 (13.76, 20.13)	<0.001 ***
Thiamine	1.00 (0.77, 1.35)	1.11 (0.83, 1.44)	0.94 (0.71, 1.27)	<0.001 ***
Riboflavin	0.46 (0.34, 0.60)	0.51 (0.38, 0.64)	0.40 (0.30, 0.55)	<0.001 *
Pyridoxine	0.38 (0.23, 0.65)	0.28 (0.17, 0.42)	0.40 (0.30, 0.55)	<0.001 ***
Vitamin C	40.80 (24.77, 67.50)	29.58 (18.03, 46.09)	55.73 (35.30, 81.68)	<0.001 ***
Niacin	10.13 (7.44, 13.28)	10.91 (8.21, 13.75)	9.12 (7.02, 12.59)	<0.001 ***
Calcium	164.00 (125.00, 223.00)	165.17 (121.92, 219.75)	163.33 (125.83, 224.17)	0.96
Potassium	1437.67 (1131.27, 1820.70)	1319.63 (1034.22, 1681.88)	1551.80 (1215.68, 2018.37)	<0.001 ***
Phosphorus	798.73 (631.73, 988.80)	822.98 (666.18, 999.98)	759.40 (590.40, 985.60)	0.065
Magnesium	260.33 (202.00, 319.67)	267.17 (211.33, 324.17)	249.33 (196.33, 315.50)	0.113
Iron	18.80 (14.37, 24.63)	19.15 (14.86, 24.57)	18.37 (13.82, 25.30)	0.471
Zinc	6.36 (5.06, 8.16)	6.68 (5.45, 8.55)	5.84 (4.65, 8.11)	0.001 **
Selenium	35.51 (26.79, 48.00)	34.50 (25.56, 44.67)	36.98 (27.41, 51.49)	<0.032 *
Food groups				
Grain	647.00 (513.33, 828.33)	627.50 (482.98, 771.95)	695.00 (542.67, 913.50)	<0.001 ***
Vegetables	80.00 (41.70, 137.00)	80.85 (36.70, 154.78)	77.50 (48.33, 125.00)	0.633
Fruits	0.00 (0.00, 0.00)	0.00 (0.00, 0.00)	0.00 (0.00, 0.00)	0.004 **
Meats	0.00 (0.00, 10.00)	0.00 (0.00, 21.70)	0.00 (0.00, 0.00)	<0.001 ***
Fish	0.00 (0.00, 0.00)	0.00 (0.00, 0.00)	0.00 (0.00, 0.00)	0.008 **
Eggs	0.00 (0.00, 20.00)	0.00 (0.00, 42.00)	0.00 (0.00, 0.00)	<0.001 ***
Dairy	0.00 (0.00, 0.00)	0.00 (0.00, 0.00)	0.00 (0.00, 0.00)	0.986
Beans	0.00 (0.00, 0.00)	0.00 (0.00, 0.00)	0.00 (0.00, 0.00)	0.001 **
Oil	23.00 (18.33, 27.67)	25.17 (20.00, 31.00)	21.00 (16.83, 24.83)	<0.001 ***

Energy, nutrients, and food intakes are expressed as median (interquartile range, IQR). Nutrient units: energy in kcal, protein, fat, carbohydrate, and food intake in g, vitamin A an equivalent in µg retinol, selenium in μg, all other nutrients in mg. *p* values were calculated by Mann–Whitney *U*-test. * *p* < 0.05, ** *p* < 0.01, *** *p* < 0.001.

**Table 3:** Intakes of energy, nutrients and food groups based on dietary diversity score groups (*N* = 499).

Nutrients	Low (*n* = 378)	Medium (*n* = 86)	High (*n* = 35)	*p*
Energy	1758.67 (1392.42, 2173.83)	1855.50 (1441.25, 2241.00)	1906.00 (1267.00, 2424.67)	0.546
Protein	48.73 (38.62, 61.44) ^a^	54.00 (44.04, 70.12) ^b^	58.50 (42.33, 78.00) ^b^	0.006 **
Fat	29.50 (23.66, 35.94) ^a^	46.53 (32.73, 61.98) ^b^	53.50 (35.03, 71.33) ^b^	<0.001 ***
Carbohydrate	317.42 (248.02, 405.75)	291.73 (231.52, 369.92)	307.13 (209.30, 371.20)	0.03 *
Cholesterol	0.00 (0.00, 26.67) ^a^	200 (112.17, 370.92) ^b^	277.33 (228.67, 508.33) ^b^	<0.001 ***
Vitamin A	27.83 (11.67, 70.00) ^a^	149.50 (86.50, 233.58) ^b^	205.67 (116.67, 253.33) ^b^	<0.001 ***
Vitamin E	17.66 (13.98, 21.39) ^a^	20.60 (16.88, 26.43) ^b^	21.89 (17.31, 30.72) ^b^	<0.001 ***
Thiamine	1.02 (0.77, 1.34)	1.00 (0.77, 1.35)	0.94 (0.71, 1.44)	0.908
Riboflavin	0.41 (0.31, 0.55) ^a^	0.55 (0.46, 0.68) ^b^	0.60 (0.47, 0.84) ^b^	<0.001 ***
Pyridoxine	0.44 (0.27, 0.72) ^a^	0.27 (0.16, 0.44) ^b^	0.21 (0.15, 0.31) ^b^	<0.001 ***
Vitamin C	41.72 (26.67, 67.67)	37.10 (24.28, 66.63)	27.57 (19.20, 71.83)	0.243
Niacin	9.72 (7.33, 12.85) ^a^	10.98 (8.53, 13.88) ^b^	11.99 (8.00, 14.04) ^b^	0.006 **
Calcium	155.33 (120.25, 202.08) ^a^	190.67 (137.00, 265.08) ^b^	217.00 (165.00, 293.67) ^b^	<0.001 ***
Potassium	1438.83 (1148.53, 1871.01)	1391.92 (1039.88, 1755.23)	1502.43 (1187.67, 1767.70)	0.679
Phosphorus	773.82 (618.58, 975.43) ^a^	833.85 (663.14, 999.23)	870.73 (642.87, 1147.23) ^b^	0.133
Magnesium	259.17 (203.42, 322.75)	265.33 (200.92, 318.75)	268.00 (183.67, 320.33)	0.945
Iron	18.67 (14.35, 24.39)	18.45 (14.79, 25.40)	22.50 (13.27, 25.53)	0.748
Zinc	6.11 (4.84, 7.93) ^a^	6.69 (5.65, 8.89) ^b^	8.09 (5.62, 10.47) ^b^	<0.001 ***
Selenium	34.70 (25.85, 45.67)	37.58 (28.86, 56.01)	40.38 (30.84, 61.47)	0.015 *
Food groups				
Grains	673.30 (542.48, 876.50) ^a^	591.00 (444. 33, 773.10) ^b^	533.30 (386.70, 645.70) ^b^	<0.001 ***
Vegetables	74.50 (33.33, 126.70) ^a^	113.35 (62.90, 181.68) ^b^	84.30 (49.00, 155.00) ^b^	<0.001 ***
Fruits	00.00 (0.00, 0.00) ^a^	0.00 (0.00, 24.17) ^b^	33.30 (0.00, 116.70) ^c^	<0.001 ***
Meats	0.00 (0.00, 0.00) ^a^	17.50 (3.33, 53.30) ^b^	38.30 (10.00, 76.70) ^b^	<0.001 ***
Fish	0.00 (0.00, 0.00) ^a^	0.00 (0.00, 0.00) ^a^	0.00 (0.00, 0.00) ^b^	<0.001 ***
Eggs	0.00 (0.00, 0.00) ^a^	31.50 (0.00, 56.15) ^b^	44.00 (22.00, 76.00) ^b^	<0.001 ***
Dairy	0.00 (0.00, 0.00) ^a^	0.00 (0.00, 0.00) ^b^	0.00 (0.00, 0.00) ^b^	0.001 **
Beans	0.00 (0.00, 0.00) ^a^	0.00 (0.00, 0.00) ^a^	0.00 (0.00, 0.00) ^b^	<0.001 ***
Oil	22.00 (18.00, 26.67) ^a^	25.00 (19.25, 30.75) ^b^	28.33 (20.67, 33.33) ^b^	<0.001 ***

Energy, nutrients, and food intakes were expressed as median (interquartile range, IQR). Nutrient units: energy in kcal, protein, fat, carbohydrate, and food intake in g, vitamin A an equivalent in µg retinol, selenium in μg, all other nutrients in mg. *p* values were obtained by Kruskal–Wallis test. ^a, b, c^ Different lowercase letters in the same row suggested a significant difference between the groups (*p* < 0.05). Kruskal–Wallis was used for multiple comparisons. * *p* < 0.05, ** *p* < 0.01, *** *p* < 0.001.

**Table 4:** Nutrient adequacy ratio of specific nutrients in two ethnicity groups (*N* = 499).

NARs	Overall (*N* = 499)	Han (*n* = 250)	Dongxiang (*n* = 249)	*p*
Vitamin A	0.088 (0.028, 0.251)	0.154 (0.061, 0.344)	0.044 (0.018, 0.126)	<0.001 ***
Vitamin E	1.302 (1.051, 1.612)	1.448 (1.107, 1.831)	1.223 (0.983, 1.438)	<0.001 ***
Thiamine	0.892 (0.692, 1.200)	0.992 (0.738, 1.262)	0.825 (0.663, 1.079)	<0.001 ***
Riboflavin	0.400 (0.308, 0.533)	0.450 (0.350, 0.558)	0.350 (0.280, 0.479)	<0.001 ***
Pyridoxine	0.315 (0.183, 0.531)	0.215 (0.131, 0.342)	0.450 (0.284, 0.700)	<0.001 ***
Vitamin C	0.482 (0.291, 0.796)	0.348 (0.212, 0.542)	0.662 (0.417, 0.968)	<0.001 ***
Niacin	0.899 (0.689, 1.143)	0.971 (0.746, 1.205)	0.824 (0.629, 1.087)	<0.001 ***
Calcium	0.231 (0.176, 0.303)	0.228 (0.167, 0.294)	0.236 (0.182, 0.306)	0.117
Potassium	0.720 (0.566, 0.915)	0.660 (0.517, 0.841)	0.789 (0.611, 1.012)	<0.001 ***
Magnesium	0.931 (0.725, 1.144)	0.955 (0.755, 1.158)	0.898 (0.702, 1.137)	0.164
Iron	1.959 (1.392, 2.689)	1.943 (1.457, 2.691)	1.970 (1.277, 2.689)	0.624
Zinc	0.738 (0.568, 0.964)	0.766 (0.592, 1.013)	0.703 (0.532, 0.885)	0.008 **
Phosphorus	1.332 (1.055, 1.664)	1.372 (1.119, 1.677)	1.267 (0.989, 1.653)	0.100
Selenium	0.713 (0.542, 0.968)	0.690 (0.511, 0.894)	0.749 (0.551, 1.031)	0.022 *
MAR	0.813 (0.648, 1.035)	0.820 (0.657, 1.053)	0.800 (0.635, 1.004)	0.399

NAR: nutrient adequacy ratio. MAR: mean adequacy ratio. *p* values were calculated by Mann–Whitney *U*-test. * *p* < 0.05, ** *p* < 0.01, *** *p* < 0.001.

**Table 5:** Nutrient adequacy ratio of specific nutrients by DDS groups (*N* = 499).

NARs	Low (*n* = 378)	Medium (*n* = 86)	High (*n* = 35)	*p* ^1^	*r* ^2^	*p*
Vitamin A	0.053 (0.022, 0.128) ^a^	0.304 (0.187, 0.455) ^b^	0.367 (0.236, 0.501) ^b^	<0.001 ***	0.708	<0.001 ***
Vitamin E	1.261 (0.999, 1.528) ^a^	1.471 (1.205, 1.888) ^b^	1.564 (1.236, 2.194) ^b^	<0.001***	0.306	<0.001 ***
Thiamine	0.892 (0.692, 1.181)	0.923 (0.704, 1.242)	0.808 (0.683, 1.200)	0.902	0.036	0.426
Riboflavin	0.367 (0.283, 0.475) ^a^	0.500 (0.415, 0.643) ^b^	0.533 (0.450, 0.700) ^b^	<0.001 ***	0.419	<0.001 ***
Pyridoxine	0.356 (0.208, 0.584) ^a^	0.219 (0.123, 0.360) ^b^	0.169 (0.115, 0.258) ^b^	<0.001 ***	−0.315	<0.001 ***
Vitamin C	0.491 (0.314, 0.797)	0.465 (0.286, 0.807)	0.324 (0.226, 0.845)	0.251	−0.072	0.108
Niacin	0.852 (0.675, 1.123) ^a^	0.988 (0.785, 1.345) ^b^	1.009 (0.729, 1.278)	0.002 **	0.207	<0.001 ***
Calcium	0.221 (0.167, 0.287) ^a^	0.272 (0.208, 0.402) ^b^	0.318 (0.232, 0.395) ^b^	<0.001 ***	0.259	<0.001 ***
Iron	1.959 (1.399, 2.644)	1.906 (1.390, 2.754)	2.274 (1.120, 2.837)	0.885	0.051	0.254
Potassium	0.720 (0.574, 0.936)	0.702 (0.526, 0.881)	0.751 (0.594, 0.884)	0.819	−0.021	0.646
Magnesium	0.926 (0.730, 1.154)	0.952 (0.730, 1.140)	0.957 (0.656, 1.144)	0.963	0.021	0.643
Zinc	0.700 (0.547, 0.901) ^a^	0.828 (0.629, 1.067) ^b^	0.940 (0.715, 1.173) ^b^	<0.001 ***	0.219	<0.001 ***
Phosphorus	1.290 (1.035, 1.636)	1.421 (1.126, 1.688)	1.451 (1.072, 1.912)	0.089	0.125	0.005 **
Selenium	0.694 (0.517, 0.914)	0.784 (0.582, 1.127)	0.808 (0.617, 1.229)	0.090	0.145	0.001 **
MAR	0.789 (0.636, 0.100) ^a^	0.891 (0.706, 1.126) ^b^	0.970 (0.666, 1.204)	0.005 **	0.170	<0.001 ***

NAR: nutrient adequacy ratio. MAR: mean adequacy ratio. ^1^ *p*-values were calculated by Kruskal–Wallis. ^2^ Spearman’s correlation coefficients (*r*) were calculated between participants’ NAR values and DDS. ^a, b^ Different lowercase letters in the same row suggested a significant difference between the groups (*p* < 0.05). SNK test was used for multiple comparisons. ** *p* < 0.01, *** *p* < 0.001.

**Table 6:** Linear regression model on predictors of MAR among smallholder farmers (*N* = 499).

Variables	*β*	95% CI	*p*
Gender			
Female (vs male)	−0.140	(−0.196, −0.083)	<0.001 ***
Nation			
Han (vs Dongxiang)	0.084	(0.014, 0.155)	0.018 *
Age			
45~ (vs 18~)	0.068	(0.006, 0.130)	0.032 *
60~ (vs 18~)	0.021	(−0.062, 0.104)	0.621
Education level			
Middle school (vs primary or below)	0.021	(−0.06, 0.101)	0.616
High school/secondary technical (vs primary or below)	0.018	(−0.094, 0.131)	0.748
College/technical school (vs primary or below)	−0.018	(−0.172, 0.136)	0.823
Household monthly income (yuan)			
1500~ (vs <1500)	0.044	(−0.023, 0.112)	0.198
2500~ (vs <1500)	0.049	(−0.052, 0.151)	0.341
≥3500 (vs <1500)	0.116	(0.012, 0.219)	0.028 *
DDS			
Medium (vs low)	0.060	(−0.013, 0.132)	0.105
High (vs low)	0.128	(0.019, 0.238)	0.021 *
Nutritional knowledge			
Above the mean (vs below the mean)	0.050	(−0.008, 0.109)	0.093
Household size			
3~ (vs <3)	0.016	(−0.091, 0.124)	0.764
6~ (vs <3)	−0.034	(−0.146, 0.078)	0.548
≥9 (vs <3)	0.024	(−0.121, 0.17)	0.745

* p < 0.05, *** *p* < 0.001.

## Data Availability

The data presented in this study are available on request from the corresponding author. The data are not publicly available due to confidentiality.

## References

[1] Zhao W., Yu K., Tan S., Zheng Y., Zhao A., Wang P., Zhang Y. (2017). Dietary diversity scores: An indicator of micronutrient inadequacy instead of obesity for Chinese children. BMC Public Health.

[2] Bailey R.L., West K.P., Black R.E. (2015). The epidemiology of global micronutrient deficiencies. Ann. Nutr. Metab..

[3] Farhat G., Lees E., Macdonald-Clarke C., Amirabdollahian F. (2019). Inadequacies of micronutrient intake in normal weight and overweight young adults aged 18-25 years: A cross-sectional study. Public Health.

[4] Otsuka R., Kato Y., Nishita Y., Tange C., Nakamoto M., Tomida M., Imai T., Ando F., Shimokata H., Suzuki T. (2016). Dietary diversity and 14-year decline in higher-level functional capacity among middle-aged and elderly Japanese. Nutrition.

[5] Meng L., Wang Y., Li T., Loo-Bouwman C.A.V., Zhang Y., Man-Yau Szeto I. (2018). Dietary Diversity and Food Variety in Chinese Children Aged 3-17 Years: Are They Negatively Associated with Dietary Micronutrient Inadequacy?. Nutrients.

[6] Liu Z., Zhao L., Man Q., Wang J., Zhao W., Zhang J. (2019). Dietary micronutrients intake status among Chinese elderly people living at home: Data from CNNHS 2010-2012. Nutrients.

[7] Harris R.M., Rose A.M.C., Forouhi N.G., Unwin N. (2020). Nutritional adequacy and dietary disparities in an adult Caribbean population of African descent with a high burden of diabetes and cardiovascular disease. Food Sci. Nutr..

[8] Hatløy A., Torheim L.E., Oshaug A. (1998). Food variety-a good indicator of nutritional adequacy of the diet? A case study from an urban area in Mali, West Africa. Eur. J. Clin. Nutr..

[9] Ito T., Tanisawa K., Kawakami R., Usui C., Ishii K., Suzuki K., Sakamoto S., Muraoka I., Oka K., Higuchi M. (2020). Micronutrient intake adequacy in men and women with a healthy Japanese dietary pattern. Nutrients.

[10] Zhang Q., Wan Q.Q., Yu S.Y., Liu Z.T., Zhao J., Li J.J., Wang X.W., Ruan Y., Wan R. (2015). Correlation of dietary diversity and growth of children aged 2~6 years old in poor areas, Yunnan. Chin. J. Child Health Care.

[11] Frempong R.B., Annim S.K. (2017). Dietary diversity and child malnutrition in Ghana. Heliyon.

[12] Chakona G., Shackleton C. (2017). Minimum dietary diversity scores for women indicate micronutrient adequacy and food insecurity status in South African towns. Nutrients.

[13] Ayogu R. (2019). Energy and nutrient intakes of rural Nigerian schoolchildren: Relationship with dietary diversity. Food Nutr. Bull..

[14] Gao Y., Zheng Z., Henneberry S.R. (2020). Is nutritional status associated with income growth? Evidence from Chinese adults. China Agric. Econ. Rev..

[15] Jiang H., Zhang J., Du W., Su C., Zhang B., Wang H. (2020). Energy intake and energy contributions of macronutrients and major food sources among Chinese adults: CHNS 2015 and CNTCS 2015. Eur. J. Clin. Nutr..

[16] Du S.F., Lu B., Zhai F.Y., Popkin B.M. (2002). A new stage of the nutrition transition in China. Public Health Nutr..

[17] Wang C., Wan G.H., Wu W.Z. (2020). Transformation of China’s poverty-reduction Strategy and Related Challenges. China Ind. Econ..

[18] Gansu Statistics Bureau. http://tjj.gansu.gov.cn/tjnj/2020/zk/indexch.htm.

[19] McGuire S. (2013). WHO, World Food Programme, and International Fund for Agricultural Development. 2012. The State of Food Insecurity in the World 2012. Economic growth is necessary but not sufficient to accelerate reduction of hunger and malnutrition. Rome, FAO. Adv. Nutr..

[20] Arimond M., Wiesmann D., Becquey E., Carriquiry A., Daniels M.C., Deitchler M., Fanou-Fogny N., Joseph M.L., Kennedy G., Martin-Prevel Y. (2010). Simple food group diversity indicators predict micronutrient adequacy of women’s diets in 5 diverse, resource-poor settings. J. Nutr..

[21] Zheng H.F., Yang J.W., Zhou H.X. Comparative on Physical Fitness Growth between Dongxiang and Rural Han Nationality 7 similar to 18 Years Students. Proceedings of the 3rd International Conference on Physical Education and Society Management (ICPESM).

[22] Su P., Zhou G., Ma h., Ma L.Y., Lei L.F., Wang N. (2017). The prevalence of dyslipidemia in middle-aged and elderly residents of the Dongxiang Chinese in Gansu province. Chin. J. Gerontol..

[23] Zhang Q., Chen X., Liu Z., Varma D.S., Wan R., Zhao S. (2017). Diet diversity and nutritional status among adults in southwest China. PLoS ONE.

[24] Jiang H., Zhao A., Zhao W., Tan S., Zhang J., Zhang Y., Wang P. (2018). Do Chinese preschool children eat a sufficiently diverse diet? A cross-sectional study in China. Nutrients.

[25] Yu D., He Y.n., Guo Q., Fang H., Xu X., Fang Y., Li J., Zhao L. (2016). Trends of energy and nutrients intake among Chinese population in 2002-2012. J. Hyg. Res..

[26] Wang Z. (2008). Atlas of the Illustrative Food Pictures for Use in Dietary Intake Recall.

[27] Bei-fan Z. (2002). Predictive values of body mass index and waist circumference for risk factors of certain related diseases in Chinese adults-study on optimal cut-off points of body mass index and waist circumference in Chinese adults. Biomed. Environ. Sci..

[28] Tian X., Xu X., Zhang K., Wang H. (2017). Gender difference of metabolic syndrome and its association with dietary diversity at different ages. Oncotarget.

[29] Gómez G., Nogueira Previdelli Á., Fisberg R.M., Kovalskys I., Fisberg M., Herrera-Cuenca M., Cortés Sanabria L.Y., Yépez García M.C., Rigotti A., Liria-Domínguez M.R. (2020). Dietary diversity and micronutrients adequacy in women of childbearing age: Results from ELANS study. Nutrients.

[30] Chinese Nutrition Society (2014). Chinese Dietary Reference Intakes (2013).

[31] Yin Z., Fei Z., Qiu C., Brasher M.S., Kraus V.B., Zhao W., Shi X., Zeng Y. (2017). Dietary diversity and cognitive function among elderly people: A population-based study. J. Nutr. Health Aging.

[32] Bi J., Liu C., Li S., He Z., Chen K., Luo R., Wang Z., Yu Y., Xu H. (2019). Dietary diversity among preschoolers: A cross-sectional study in poor, rural, and ethnic minority areas of central south China. Nutrients.

[33] Liu J., Shively G.E., Binkley J.K. (2014). Access to variety contributes to dietary diversity in China. Food Policy.

[34] Bezerra I.N., Sichieri R. (2011). Household food diversity and nutritional status among adults in Brazil. Int. J. Behav. Nutr. Phys. Act..

[35] Mirmiran P., Azadbakht L., Esmaillzadeh A., Azizi F. (2004). Dietary diversity score in adolescents—a good indicator of the nutritional adequacy of diets: Tehran lipid and glucose study. Asia Pac. J. Clin. Nutr..

[36] Miller V., Yusuf S., Chow C.K., Dehghan M., Corsi D.J., Lock K., Popkin B., Rangarajan S., Khatib R., Lear S.A. (2016). Availability, affordability, and consumption of fruits and vegetables in 18 countries across income levels: Findings from the Prospective Urban Rural Epidemiology (PURE) study. Lancet Glob. Health.

[37] Singh S., Jones A.D., Jain M. (2020). Regional differences in agricultural and socioeconomic factors associated with farmer household dietary diversity in India. PLoS ONE.

[38] Desta M., Akibu M., Tadese M., Tesfaye M. (2019). Dietary diversity and associated factors among pregnant women attending antenatal clinic in Shashemane, Oromia, Central Ethiopia: A cross-sectional study. J. Nutr. Metab..

[39] Jones A.D. (2017). Critical review of the emerging research evidence on agricultural biodiversity, diet diversity, and nutritional status in low-and middle-income countries. Nutr. Rev..

[40] Ochieng J., Afari-Sefa V., Lukumay P.J., Dubois T. (2017). Determinants of dietary diversity and the potential role of men in improving household nutrition in Tanzania. PLoS ONE.

[41] Fukuda Y., Ishikawa M., Yokoyama T., Hayashi T., Nakaya T., Takemi Y., Kusama K., Yoshiike N., Nozue M., Yoshiba K. (2017). Physical and social determinants of dietary variety among older adults living alone in Japan. Geriatr. Gerontol. Int..

[42] Otto M.C., Padhye N.S., Bertoni A.G., Jacobs D.R., Mozaffarian D. (2015). Everything in Moderation-Dietary Diversity and Quality, Central Obesity and Risk of Diabetes. PLoS ONE.

[43] Tesfaye T.S., Zeleke T.M., Alemu W., Argaw D., Bedane T.K. (2020). Dietary diversity and physical activity as risk factors of abdominal obesity among adults in Dilla town, Ethiopia. PLoS ONE.

[44] Oldewage-Theron W.H., Kruger R. (2008). Food variety and dietary diversity as indicators of the dietary adequacy and health status of an elderly population in Sharpeville, South Africa. J. Nutr. Elder..

[45] Tavakoli S., Dorosty-Motlagh A.R., Hoshiar-Rad A., Eshraghian M.R., Sotoudeh G., Azadbakht L., Karimi M., Jalali-Farahani S. (2016). Is dietary diversity a proxy measurement of nutrient adequacy in Iranian elderly women?. Appetite.

[46] Quann E.E., Fulgoni V.L., Auestad N. (2015). Consuming the daily recommended amounts of dairy products would reduce the prevalence of inadequate micronutrient intakes in the United States: Diet modeling study based on NHANES 2007-2010. Nutr. J..

[47] Masset G., Scarborough P., Rayner M., Mishra G., Brunner E.J. (2015). Can nutrient profiling help to identify foods which diet variety should be encouraged? Results from the Whitehall II cohort. Br. J. Nutr..

[48] Zhao L.Y., Fang Y.H., He Y.N., Yu D.M., Guo Q.Y., Yu W.T., Fang H.Y., Wang X., Zhao W.H. (2016). Trends of food consumption among Chinese population in 1992—2012. J. Hyg. Res..

[49] Carr A.C., Maggini S. (2017). Vitamin C and Immune Function. Nutrients.

[50] Cano-Ibáñez N., Gea A., Martínez-González M.A., Salas-Salvadó J., Corella D., Zomeño M.D., Romaguera D., Vioque J., Aros F., Wärnberg J. (2019). Dietary diversity and nutritional adequacy among an older Spanish population with metabolic syndrome in the PREDIMED-Plus study: A cross-sectional analysis. Nutrients.

[51] Gavelle E., Huneau J.F., Mariotti F. (2018). Patterns of protein food intake are associated with nutrient adequacy in the general French adult population. Nutrients.

